# Scientific migration of junior scientists to China

**DOI:** 10.1186/gb4180

**Published:** 2014-06-24

**Authors:** Ling-Ling Chen

**Affiliations:** 1 State Key Laboratory of Molecular Biology, Institute of Biochemistry and Cell Biology, Shanghai Institutes for Biological Sciences, Chinese Academy of Sciences, 320 Yueyang Road, Shanghai 200031, China

## 

As a junior investigator in biology who recently set up a laboratory in Shanghai, China, I frequently get asked why I chose to start a laboratory in China, how I feel running a laboratory there and what are the main challenges I face. People who asked me such questions are not only established researchers but also postdoctoral fellows and senior PhD students. Interestingly, most of these junior scientists are originally from China but trained in the West and now are ready to move back to their homeland.

About 10 years ago I took my first international flight from Shanghai to the USA to pursue graduate study. Although I was well aware of the cultural differences between the two countries, it took some time to adjust to living in Connecticut, both socially and academically: these differences include not only the smaller everyday matters of life such as food, language and manner, but also the way that people in different countries view the world. I ended up as a PhD student with Dr Gordon Carmichael, who has been tremendously supportive for my career development. Over a period of 7 years I obtained both a PhD and an MBA degree, finished a short period of postdoctoral training, got research funding and received a promotion to Assistant Professor in Residence at the University of Connecticut. During these 7 years in Connecticut, however, much also changed in China, especially national support for research funding and the emergence of a more rigorous academic environment.

Research funding for fundamental science, such as biology, has significantly increased over the last 10 years. This has led to many and better job opportunities for junior investigators in China. Also, the amount of funding for each grant has also been dramatically raised. For example, a general grant from the National Natural Science Foundation of China is about ¥800,000 (US$120,000)/4 years now, compared with ¥200,000 ($30,000)/4 years only a few years ago. In addition, the grant application process and decision making have become more transparent and fair. Generally, if you are doing good research, you can now expect to be funded - more or less - and this support is at a level that will allow you to continue key projects you are working on. While ‘good research’ is still largely judged by the publication of your results in ‘high-impact’ journals, this is not really much different than the situation elsewhere. Academically, research in China is becoming more sustainable. For instance, novel research is expected and a tenure or tenure-like system has been launched recently in many institutions and universities. Thus, the quality of research is emphasized in addition to the number of publications. However, although total research funding has increased, it is worth noting that grant applications are becoming more and more competitive than years ago, due to the fact that a large number of scientists have recently migrated to China and are now competing for these funds. Thus, now it is time for us to continue this trend by encouraging China to commit to increased and sustained research investment.

These exciting changes over the last 10 years have made the research environment in China, especially that in large cities, similar to that found in the West. The boom in research funding has provided more opportunities to allow well-trained young scientists to get jobs and also for them to continue their research in China. As a biologist, I would say, these changes are the most important factors that attracted me to return.

Well, if you like these changes, you may also want to list China as one of the places to send out job applications to. To me, running a laboratory in China is similar to that in other places; although moving back to China after living abroad for many years, I have surprisingly but inevitably experienced a ‘reverse culture shock’. For example, the popular application of a new Chinese cyber language or text speak really challenged me. Ultimately, running a laboratory is very much like managing a small business where you need to generate ideas, raise money and recruit the right team to work with you. I am sure that anyone who is ready to open business has no problem getting ideas; so the real questions are where do you get money and who will best help you to realize these ideas?

## Start-up packages and funding

The start-up packages are generous in most research units in China compared with 10 years ago. In addition to institutional and regional support, several types of national awards have been funded to attract well-trained people to return to China. Added up, the start-up packages for junior faculty members are comparable to those in America. In addition, many junior scientists are likely to get grants from the National Natural Science Foundation of China in their early career years, simply with creative ideas and careful proposal preparation.

## People

Compared with salaries paid in the West, as well as in other places in Asia, such as Singapore and Japan, laborers (including faculty members) are paid less in China. Therefore, it is difficult to recruit qualified technicians and postdoctoral fellows. However, lower salaries allow us to easily build up relatively large-sized research teams. A great strength in many research units in China is the quality of graduate students, who are talented, self-motivated and dedicated. However, recruiting graduate students is getting more and more difficult since many new laboratories have been established in the past several years, while the total number of graduate students has remained largely unchanged in many research institutes. Importantly, it is the faculty’s responsibility to train graduate students/technicians/postdoctoral fellows and maintain the team moving forward in the first several years.

## Equipment and reagents

The equipment and facilities installed in many research units in China are state-of-the-art, and most of them are readily available to scientists. If you can run them effectively, then you are very welcome to work with them. In addition to many domestic chemical and biological companies, almost all big international companies now have distributors in China. One important issue is that while it is easy to place an order for a reagent from a foreign country, such orders are usually more expensive owing to import taxes, agent commissions and other issues. Also, it usually takes extra days (sometimes several weeks) for shipping and handling. Therefore, many international orders must be placed a long time before the reagent is required for use, in order to keep experiments running efficiently.

Once independent careers have been set up, junior investigators face challenges in their early careers that are similar to those in other places. For example, can my research continue to be funded? Can I survive and get tenured? Ultimately, can my research be recognized in the field? Although these questions are asked by almost all junior investigators, no matter where they are located physically, there are several things that distinguish research in China from that carried out elsewhere.

## Doing your research

When you arrive back in China after training abroad, you are welcomed by large grants, awards and titles. It is easy to enjoy the attention but this can easily distract you from the important benchwork that led to your recruitment. I prefer to focus closely on my laboratory. Working in your own laboratory as a super postdoctoral fellow is probably the best way to thrive for the first several years. A super postdoctoral fellow is one that works hard daily in front of the bench, trains team members (most of them are fresh graduate students) the best way they are able, and publishes as much as they can as an independent researcher.

## Research niches

Identify a clear research focus that provides you with a niche that can lead to discoveries and publications, but keep in mind that the biological sciences are becoming much more interdisciplinary. Focus first on your existing strengths and do your best with what you are already good at. Meanwhile, it is also important to think of a few interesting questions that may need you to collaborate with other scientists.

## Communication

Although working in China, you are the part of the world, and it is very important to communicate what you are doing with others in your field. Attending international meetings regularly (although the international flight often takes a really long time!) and interacting with both peers and seniors in your field may help to let others know who you are and what your research is about. Frequently, international meetings are held in China, such as Cold Spring Harbor Asia Conferences. So, even without traveling long distances, you still have great opportunities to get together with people in your field. Besides these meeting activities, many journals, including Cell Press journals and *Genome Biology* recently visited different laboratories in different institutes/universities in China. This is mutually beneficial for both the journals and for us here.

## Recognition

There are increasing numbers of scientists who became highly recognized in their fields after returning and setting up their laboratories here. Therefore, don’t worry about whether you and your research will be recognized when you work in China - as long as you are doing a good job.

Collectively, I think there is not too much difference between running a scientific laboratory in China or anywhere else. However, many other non-scientific factors, such as family, culture, environment and language, count for a lot when choosing where to start. If China is committed to sustained and increased scientific investment, the migration of junior scientists to the rapidly growing scientific community in China will be increased. Fingers crossed!

